# Role of *cpxA* Mutations in the Resistance to Aminoglycosides and β-Lactams in *Salmonella enterica* serovar Typhimurium

**DOI:** 10.3389/fmicb.2021.604079

**Published:** 2021-02-04

**Authors:** Wenxian Jing, Juan Liu, Shanshan Wu, Xuerui Li, Yongsheng Liu

**Affiliations:** State Key Laboratory of Veterinary Etiological Biology, Lanzhou Veterinary Research Institute, Chinese Academy of Agricultural Sciences, Lanzhou, China

**Keywords:** *S. enterica* serovar Typhimurium, various mutants, resistance, AGAs, β-lactams, *cpxA*

## Abstract

Although it has been reported that deletion of the response regulator, CpxR, in the CpxRA system confers sensitivity to aminoglycosides (AGAs) and β-lactams in *Salmonella enterica* serovar Typhimurium, the regulatory effects of CpxA on multidrug resistance (MDR) are yet to be fully investigated in this organism. Here, to explore the role of CpxA in MDR, various *cpxA* mutants including a null mutant (JSΔ*cpxA*), a site-directed mutant (JSΔ*cpxA*_38_) and an internal in-frame deletion mutant (JSΔ*cpxA_92__–__104_*) of the *S. enterica* serovar Typhimurium strain JS, were constructed. It was revealed that *cpxA* and *cpxR* deletion mutants have opposing roles in the regulation of resistance to AGAs and β-lactams. Amikacin and cefuroxime can activate the CpxRA system, which results in increased resistance of the wild-type compared with the *cpxR* deletion mutant. All the *cpxA* mutations significantly increased resistance to AGAs and β-lactams due to CpxRA system activation *via* the phosphorylation of CpxR. Moreover, AckA-Pta-dependent activation of CpxR increased the antibiotic resistance of *cpxA* deletion mutants. Further research revealed that the AcrAB-TolC conferred resistance to some AGAs and β-lactams but does not influence the regulation of resistance by CpxRA against these antibiotics. The detection of candidate MDR-related CpxR regulons revealed that the mRNA expression levels of *spy*, *ycca*, *ppia*, *htpX*, *stm3031*, and *acrD* were upregulated and that of *ompW* was downregulated in various *cpxA* mutants. Furthermore, the expression levels of *nuoA* and *sdhC* mRNAs were downregulated only in JSΔ*cpxA_92__–__104_*. These results suggested that *cpxA* mutations contribute to AGAs and β-lactams resistance, which is dependent on CpxR.

## Introduction

With the overuse and misuse of antibacterial agents, the emergence and spread of multidrug resistant (MDR) *Salmonella enterica* serovar Typhimurium (*S*. Typhimurium) has become a clinical and public health threat worldwide (Levy, 2002; Quinn et al., 2006). To adapt to adverse environmental conditions, bacteria have developed many sophisticated signal transduction systems, referred to as two-component systems (TCSs). These systems sense changes in environmental pH, ion concentrations, nutrient levels, quorum signals, and antibiotic concentrations and, in response, regulate the expression of genes involved in processes such as cell growth, virulence, biofilm formation, quorum sensing, and antibiotic resistance (De la Cruz et al., 2015; Padilla-Vaca et al., 2017; Tiwari et al., 2017; Fujimoto et al., 2018). TCSs have been identified as potential drug targets. Unlike conventional antibiotics, these drugs would likely be effective against various drug-resistant bacteria and avoid the emergence of resistant strains (Gotoh et al., 2010; Tiwari et al., 2017). Understanding the regulation mechanisms of TCSs to conventional antibiotic resistance would be beneficial for the reasonable application and low occurrence of drug resistance to these TCSs-targeted antibacterial agents.

The CpxRA system is an important TCS in *Escherichia coli* and *Salmonella*. It consists of an inner membrane-bound histidine sensor kinase, CpxA, and a cytoplasmic response regulator, CpxR (De Wulf et al., 2002). CpxP is a periplasmic chaperone that binds to CpxA and inhibits its kinase activity. The periplasmic protease DegP can disrupt CpxP binding to misfolded proteins (Isaac et al., 2005). Upon sensing extracytoplasmic stresses, CpxP binds to misfolded proteins, dissociates from CpxA, and is degraded by DegP, and then CpxA autophosphorylates and donates its phosphate group to CpxR at D51 (D51). This allows phosphorylated CpxR (CpxR-P) containing a specific DNA-binding site (M199) to bind to the promoter regions of target genes at the consensus sequence (GTAAAN_5_GTAAA) (De Wulf et al., 2002; DiGiuseppe and Silhavy, 2003), and to regulate a series of target genes associated with antibiotic resistance and pathogenesis (Nishino et al., 2010; Dbeibo et al., 2018). *cpxA* knock-out mutation can result in constitutive activation of the CpxRA system. This is because *cpxA* deletion abolishes its phosphatase activity for CpxR-P, CpxR then accepts a phosphoryl group from acetyl phosphate, the product of the phosphotransacetylase (Pta)-acetate kinase (AckA) pathway (Wolfe et al., 2008). Similarly, a strain containing the mutant *cpxA* gene (*cpxA*^∗^) encoding a CpxA variant lacking several amino acids in the periplasmic loop, exhibits constitutive CpxRA system activity. This occurs because the mutant CpxA^∗^ protein still functions as an autokinase and CpxR kinase but lacks phosphatase activity, resulting in accumulation of CpxR-P and hyperactivation of the CpxRA response (Raivio and Silhavy, 1997).

The CpxRA envelope stress response system is closely related to drug resistance. In *E. coli*, *cpxA* deletion or *cpxA*^∗^ mutations will confer the resistance to hydroxyurea, β-lactams, aminoglycosides, and fosfomycin (Mahoney and Silhavy, 2013; Masi et al., 2020). Moreover, overexpression of *cpxR* can confer the resistance to kanamycin, amikacin, deoxycholate, novobiocin, and β-lactams (Hirakawa et al., 2003a,b). In *S*. Typhimurium, the *cpxRA* deletion confers susceptibility to ceftriaxone by influencing the expression of *stm1530* and *ompD* (Hu et al., 2011), and overexpression of *cpxR* in the *cpxR* deletion mutant increases resistance to AGAs and β-lactams as a result of the downregulation of outer membrane protein gene expression (Huang et al., 2016). However, the regulatory effect of CpxA on MDR has not been fully investigated. One study reported that both the *cpxR* and *cpxA* mutants were more sensitive to amikacin than the wild-type strain, but the *cpxA*^∗^ mutation renders *S*. Typhimurium more resistant to the antibiotic amikacin (Humphreys et al., 2004), which suggests that different mutant versions of the CpxA sensor protein exhibit unexpected resistance regulation in *S*. Typhimurium. Another study reported that the various activation pathways of the CpxRA system determine different sensitivities to β-lactams (Delhaye et al., 2016). Therefore, the various mutations of *cpxA* should be analyzed in concert to understand the full extent of the effects of CpxA on MDR. In this study, we constructed different mutants of the *cpxA* gene to systematically analyze the regulatory effects of CpxA on AGAs and β-lactams resistance and explored the related molecular mechanism. The results suggested that various *cpxA* mutations can activate the CpxRA system and confer resistance to AGAs and β-lactams.

## Materials and Methods

### Bacterial Strains, Plasmids, and Culture Conditions

The bacterial strains and plasmids used in this study are listed in Table 1. All bacterial strains were mutant derivatives of *S. enterica* serovar Typhimurium strain JS. All strains were cultured aerobically in Luria–Bertani (LB) medium [1% (w/v) tryptone, 0.5% (w/v) yeast extract, and 1% (w/v) NaCl, pH = 7.0] (Takara Bio., Kusatsu, Japan) at 37°C. When necessary, LB medium were supplemented with ampicillin (100 μg/ml), kanamycin (50 μg/ml), chloramphenicol (25 μg/ml), or sucrose (8%, w/v).

**TABLE 1:** Strains and plasmids used in this study.

**Strain or plasmid**	**Description**	**Reference or sources**
Strains		
JS	*S. enterica* serovar Typhimurium CVCC541	China Veterinary Culture Collection Center
JSΔ*cpxA*	Derivative of JS that lacks c*pxA*	This study
JSΔ*cpxA-CL*	Complemented strain of JSΔ*cpxA*	This study
JSΔ*cpxR*	Derivative of JS that lacks c*pxR*	This study
JSΔ*cpxRA*	Derivative of JS that lacks c*pxRA*	This study
JSΔ*ackA-pta*	Derivative of JS that lacks *ackA-pta*	This study
JSΔ*cpxA*Δ*ackA-pta*	Derivative of JS that lacks *cpxA* and *ackA-pta*	This study
JS*cpxR*_*D*__51A_	Derivative of JS with phosphorylation site mutation on the CpxR	Jing et al., 2020
JS*cpxR*_*M*__199A_	Derivative of JS with DNA-binding site mutation on the CpxR	Jing et al., 2020
JSΔ*cpxAR*_*D*__51A_	Derivative of JSΔ*cpxA* with phosphorylation site mutation on the CpxR	This study
JSΔ*cpxAR*_*M*__199A_	Derivative of JSΔ*cpxA* with DNA-binding site mutation on the CpxR	This study
JSc*pxA*_38_	Derivative of JS with L38F site-directed mutation on the CpxA	Jing et al., 2020
JSc*pxA*_92__–__104_	Derivative of JS with a deletion removing amino acids 92 to 104 of CpxA	Jing et al., 2020
JSc*pxA*_38_-CL	Complemented strain of JSc*pxA*_38_	This study
JSc*pxA*_92__–__104_-CL	Complemented strain of JSc*pxA*_92__–__104_	This study
JSc*pxA*_38_Δ*cpxR:Kan*	Derivative of JS*CpxA*_38_ that lacks c*pxR* replaced by *Kan*	This study
JSc*pxA*_92__–__104_Δ*cpxR:Kan*	Derivative of JS*CpxA*_92__–__104_ that lacks c*pxR* replaced by *Kan*	This study
JSΔ*acrB*	Derivative of JS that lacks Δ*acrB*	This study
JSΔ*acrB-CL*	Complemented strain of JSΔ*acrB*	This study
JSΔ*cpxA*Δ*acrB*	Derivative of JSΔc*pxA* that lacks *acrB*	This study
JSc*pxA*_38_Δ*acrB*	Derivative of JS*cpxA*_38_ that lacks *acrB*	This study
JSc*pxA*_92__–__104_Δ*acrB*	Derivative of JSc*pxA*_92__–__104_ that lacks *acrB*	This study
JSΔ*cpxR*Δ*acrB*	Derivative of JSΔc*pxR* that lacks *acrB*	This study
JSΔ*tolC*	Derivative of JS that lacks *tolC*	This study
JSΔ*tolC-CL*	Complemented strain of JSΔ*tolC*	This study
JSΔ*cpxA*Δ*tolC*	Derivative of JSΔc*pxA* that lacks *tolC*	This study
JSc*pxA*_38_Δ*tolC*	Derivative of JSc*pxA*_38_ that lacks *tolC*	This study
JSc*pxA*_92__–__104_Δ*tolC*	Derivative of JSc*pxA*_92__–__104_ that lacks *tolC*	This study
JSΔ*cpxR*Δ*tolC*	Derivative of JSΔc*pxR* that lacks *TolC*	This study
JSΔ*ramA::Kan*	Derivative of JS that lacks *ramA* replaced by *Kan*	This study
JSΔ*cpxA*Δ*ramA::Kan*	Derivative of JSΔ*cpxA* that lacks *ramA* replaced by *Kan*	This study
JSΔ*stm3031::Kan*	Derivative of JS that lacks *stm3031* replaced by *Kan*	This study
JSΔ*cpxA*Δ*stm3031::Kan*	Derivative of JSΔ*cpxA* that lacks *stm3031* replaced by *Kan*	This study
JSΔ*htpX::Kan*	Derivative of JS that lacks *htpX* replaced by *Kan*	This study
JSΔ*cpxA*Δ*htpX::Kan*	Derivative of JSΔ*cpxA* that lacks *htpX* replaced by *Kan*	This study
JSΔ*spy::Kan*	Derivative of JS that lacks *spy* replaced by *Kan*	This study
JSΔ*cpxA*Δ*spy::Kan*	Derivative of JSΔ*cpxA* that lacks *spy* replaced by *Kan*	This study
JSΔ*acrD::Kan*	Derivative of JS that lacks *acrD* replaced by *Kan*	This study
JSΔ*cpxA*Δ*acrD::Kan*	Derivative of JSΔ*cpxA* that lacks *acrD* replaced by *Kan*	This study
JSΔ*acrB*Δ*acrD::Kan*	Derivative of JSΔ*acrB* that lacks *acrD* replaced by *Kan*	This study
JSΔ*cpxA*Δ*acrB*Δ*acrD::Kan*	Derivative of JSΔ*cpxA*Δ*acrB* that lacks *acrD* replaced by *Kan*	This study
Plasmids		
pKD4	Template plasmid containing the kanamycin cassette, Ap^*R*^	Datsenko and Wanner, 2000
pKD46	Template plasmid containing Red recombinase system under arabinose-inducible promoter, Ap^*R*^	Datsenko and Wanner, 2000
pCP20	Template plasmid expressing FLP recombinase, Ap^*R*^, Cm^*R*^	Datsenko and Wanner, 2000
psacBKan	Derived from pSTV28 by inserting *sacB* and *Kan* gene, Cm^*r*^	Jing et al., 2020
pSTV28	Low-copy clone vector: Cm^*R*^	Takara

### Construction of Various Mutants of *S*. Typhimurium

All deletion mutants were generated *via* the Red recombinase system, as reported previously (Datsenko and Wanner, 2000). The respective primers are shown in Supplementary Table 1. The genes *cpxA*, *cpxR*, *cpxRA*, *ackA-pta*, *acrB*, and *tolC* were replaced with a kanamycin (*kan*) resistance cassette in the *S*. Typhimurium strain CVCC541, generating mutant strains JSΔ*cpxA::kan*, JSΔ*cpxR::kan*, JSΔ*cpxRA:kan*, JSΔ*ackA-pta::kan*, JSΔ*acrB::kan*, and JSΔ*tolC::kan*, respectively. Using helper plasmid pCP20, expressing the FLP recombinase, the *kan* resistance cassette was excised from the JSΔ*cpxA::kan*, JSΔ*cpxR::kan*, JSΔ*cpxRA::kan*, JSΔ*ackA-pta::kan*, JSΔ*acrB::kan*, and JSΔ*tolC::kan* mutants, generating mutant strains JSΔ*cpxA*, JSΔ*cpxR*, JSΔ*cpxRA*, JSΔ*ackA-pta*, JSΔ*acrB*, and JSΔ*tolC*, respectively. Multiple additional mutants were then constructed from these mutants using the Red recombinase system. From the JSΔ*cpxA* mutant, additional mutant strains JSΔ*cpxA*Δ*ackA-pta*, JSΔ*cpxA*Δ*acrB*, and JSΔ*cpxA*Δ*tolC* were constructed. From the JSΔ*cpxR* strain, additional mutants JSΔ*cpxR*Δ*acrB* and JSΔ*cpxR*Δ*tolC* were constructed. The main regulon member deletion mutants JSΔ*stm3031::Kan*, JSΔ*htpX::Kan*, and JSΔ*spy::Kan* were constructed from the wild-strain JS, and JSΔ*cpxA*Δ*stm3031::Kan*, JSΔ*cpxA*Δ*htpX::Kan*, and JSΔ*cpxA*Δ*spy::Kan* were constructed from the JSΔ*cpxA* mutant. The mutants JSΔ*acrD::Kan*, JSΔ*cpxA*Δ*acrD::Kan*, JSΔ*acrB*Δ*acrD::Kan*, and JSΔ*cpxA*Δ*acrB*Δ*acrD::Kan* were constructed from the corresponding strain JS, JSΔ*cpxA*, JSΔ*acrB*, and JSΔ*cpxA*Δ*acrB*.

The JS*cpxR*_*D51A*_, JS*cpxR*_*M199A*_, JS*cpxA*_38_, and JS*cpxA*_92__–__104_ mutants were constructed in our previous study (Jing et al., 2020). Subsequently, mutant strains JSΔc*pxAcpxR*_*D51A*_, JSΔc*pxAcpxR*_*M199A*_, JSΔ*cpxA*_38_*cpxR::kan*, and JSΔ*cpxA*_92__–__104_*cpxR::kan* were constructed from mutant strains JS*cpxR*_*D51A*_, JSc*pxR*_*M199A*_, JS*cpxA*_38_, and JS*cpxA*_92__–__104_, respectively, using the Red recombinase system.

### Complementation of Various Mutants

To be able to construct the complemented strains of various *cpxA* mutants (JSΔ*cpxA*, JS*cpxA*_38_, and JS*cpxA*_92__–__104_), a strategy to construct the complemented strain in the bacterial chromosome was used according to our previous study (Jing et al., 2020). In order to distinguish *cpxA* complemented strains from wild strains, we changed the terminate code “UAA” of the *cpxA* gene in the wild strain to “UAG” in the *cpxA* complemented strains. First, the *sacBKan* fragment was amplified from plasmid psacBKan using the primers CpxA-H1P1/H2P2, and then integrated into the corresponding *cpxA* mutations locus using the Red recombinase system to obtain the intermediate strain. Positive clones were screened by PCR using primer pairs CpxA-F/R, CpxA-F/sacBKan-k2, sacBKan-k1/CpxA-R, and sacBKan-k1/k2. Then, using primers CpxA-UF/CpxA-CL-UR and CpxA-CL-DF/CpxA-DR, the wild-type *cpxA* fragment with synonymous substitution of terminates codon was prepared on the *Bam*HI-digested vector pSTV28. The recombinogenic *cpxA* fragment was amplified using primer pair CpxA^∗^-F/R. Finally, the recombinogenic *cpxA* fragment replaced the *sacBKan* cassette and the corresponding *cpxA* complemented strains (JSΔ*cpxA-CL*, JS*cpxA_38_-CL*, and JS*cpxA*_92__–__104_-*CL* ) were confirmed by PCR.

The *acrB* and *tolC* complemented strains in the chromosome were constructed using the λ Red recombination system (Datsenko and Wanner, 2000). The corresponding PCR products were obtained using primer pairs AcrB-CL-F/AcrB-CL-R and TolC-CL-F/TolC-CL-F. To distinguish *acrB* complemented strains from wild strains, we changed the 884th codon “GTC” of the *acrB* gene in the wild strain to “GTG” in the *acrB* complemented strains. To distinguish *tolC* complemented strains from wild strains, we changed the terminate code “UGA” of the *tolC* gene in the wild strain to “UAA” in the *tolC* complemented strains. The corresponding PCR products were then used to replace the deletion mutations using the λ Red recombinase system. The complemented strains (JSΔ*acrB-CL* and JSΔ*tolC-CL*) were selected on LB agar plates containing cefuroxime (1 μg/ml) and confirmed by Sanger sequencing.

### Real-Time Quantitative PCR (qPCR) Analysis of Bacterial Gene Expression

Total RNA was isolated from bacterial cultures (OD_600_ = 1.0) using a TransZol UP Plus RNA Kit (TransGen Biotech, Beijing, China) according to the manufacturer’s instructions. Aliquots (1 μg) of the total RNA were then transcribed into cDNA using a PrimeScript RT Reagent Kit with gDNA Eraser (Takara Bio., Kusatsu, Japan). Quantitative PCR (qPCR) assays were performed using the CFX96 Real-Time System and a C1000 Touch Thermal Cycler (Bio-Rad, Hercules, CA, United States), as well as a QuantiNova SYBR Green PCR Kit (Qiagen, Hilden, Germany). The primers used for qPCR analysis are outlined in Supplementary Table 1. The expression levels of all genes were normalized against that of internal reference gene *rpoD*, and relative fold changes in expression were calculated using the 2^–ΔΔ*CT*^ method. The expression level of each mRNA in strain JS represents a one-fold change.

### Antibiotic-Induced Activation Assays

Briefly, the test strains were grown overnight in LB medium, diluted 1:100, and subcultured to the mid-exponential phase (OD_600_ of 0.4 to 0.6). When we used LB medium for the antibiotic activation test, it was found that the MICs values of the JS strain against amikacin and cefuroxime was 16 and 8 μg/ml in LB, respectively. So, to observe the relationship between the concentration of amikacin and cefuroxime with the activation level of the CpxRA system, the *S*. Typhimurium wild-type strain was grown in LB medium or LB medium with 4, 8, or 16 μg/ml amikacin or 1, 2, or 4 μg/ml cefuroxime to a stationary growth phase (OD_600_ ≥ 1.0). To observe the influence of Δ*cpxA* or Δ*cpxR* on induction of amikacin and cefuroxime, the wild-type, Δ*cpxA*, and Δ*cpxR* cells were grown in LB medium with 16 μg/ml amikacin or 4 μg/ml cefuroxime to a stationary growth phase (OD_600_ ≥ 1.0). The mRNA expression levels of the target genes *cpxP* and *degP* were used to measure the activation level of the CpxRA system.

### Antibiotic Susceptibility Testing

The MIC values of selected antibiotics against the various bacterial strains were measured in Mueller-Hinton (MH) medium (Hopebio, Qingdao, China) *via* the broth microdilution method as per the Clinical and Laboratory Standards Institute’s (CLSI) guidelines (Cockerill et al., 2012). The tested antibiotics included amikacin (AMK), netilmicin (NET), streptomycin (STR), kanamycin (KAN), cefalotin (CEP), cefuroxime (CXM), ceftriaxone (CRO), and cefotaxime (CTX). All assays were conducted in triplicate.

### Statistical Analysis

Statistical analyses were performed using GraphPad Prism 6.0 Windows software and data were compared using the Student’s *t*-test (GraphPad Software, United States). A probability (*p*) value of <0.05 was considered statistically significant.

## Results

### *cpxA* and *cpxR* Deletion Mutants Have Opposing Roles in the Regulation of Resistance to AGAs and β-Lactams

As described in the introduction, *cpxA* deletion mutations sometimes show different resistance to amikacin. To investigate the effects of *cpxA* deletion mutations on the regulation of drug resistance, *cpxA* and *cpxR* deletion mutants – JSΔ*cpxA* and JSΔ*cpxR* – were generated from the *S*. Typhimurium strain JS. The complemented strain JSΔ*cpxA-CL* was also prepared. As shown in Table 2, compared with parental strain JS, two- to eight-fold increases in the MICs of the tested AGA and β-lactam antibiotics against the *cpxA* null mutant were observed; whereas two- to eight-fold decreases in the MICs of the tested AGA and β-lactam antibiotics against the *cpxR* deletion mutant were observed. The MICs of AGA and β-lactam antibiotics decreased by two- to eight-fold for the complemented strain JSΔ*cpxA-CL*, as compared to those for JSΔ*cpxA*. These results indicated that deletions of *cpxA* and *cpxR* have opposing roles in the regulation of AGAs and β-lactams resistance.

**TABLE 2:** Susceptibility of *S. enterica* serovar Typhimurium to several AGA and β-lactam antibiotics.

**Strain**	**MICs (μg/mL)**							
	**AMK**	**NET**	**STR**	**KAN**	**CEP**	**CXM**	**CRO**	**CTX**
JS	2	0.25	16	2	4	4	0.0625	0.0625
JSΔ*cpxA*	8	0.5	32	8	32	16	0.25	0.25
JSΔ*cpxA-CL*	2	0.25	16	2	4	4	0.0625	0.0625
JSΔ*cpxR*	0.5	0.0625	2	1	2	2	0.03125	0.03125
JSΔ*cpxRA*	0.5	0.0625	2	1	2	2	0.03125	0.03125
JSΔ*acka-pta*	4	0.25	16	4	2	4	0.125	0.03125
JSΔ*cpxA*Δ*acka-pta*	4	0.25	16	4	2	4	0.125	0.03125
JS*cpxR*_*D*__51A_	1	0.0625	4	1	2	2	0.03125	0.03125
JS*cpxR*_*M*__199A_	2	0.25	16	2	4	4	0.0625	0.0625
JSΔ*cpxAR*_*D*__51A_	2	0.125	8	1	2	2	0.03125	0.03125
JSΔ*cpxAR*_*M*__199A_	2	0.125	8	1	2	4	0.0625	0.0625
JSc*pxA*_38_	8	0.5	32	8	32	16	0.25	0.25
JSc*pxA*_92__–__104_	8	0.5	32	8	32	16	0.25	0.25
JSc*pxA*_38_-CL	2	0.25	16	2	4	4	0.0625	0.0625
JSc*pxA*_92__–__104_-CL	2	0.25	16	2	4	4	0.0625	0.0625
JSc*pxA*_38_Δ*cpxR:Kan*	0.5	0.0625	2	–	2	2	0.03125	0.03125
JSc*pxA*_92__–__104_Δ*cpxR:Kan*	0.5	0.0625	2	–	2	2	0.03125	0.03125
JSΔ*acrB*	0.5	0.25	4	2	0.5	0.125	0.0625	0.03125
JSΔ*acrB-CL*	2	0.25	16	2	4	4	0.0625	0.0625
JSΔ*cpxA*Δ*acrB*	2	1	16	8	1	0.5	0.25	0.0625
JSc*pxA*_38_Δ*acrB*	4	0.5	16	8	1	0.25	0.25	0.125
JSc*pxA*_92__–__104_Δ*acrB*	4	1	16	8	1	0.25	0.25	0.125
JSΔ*cpxR*Δ*acrB*	0.125	0.0625	2	1	0.5	0.0625	0.03125	0.0157
JSΔ*tolC*	0.5	0.25	4	1	0.5	0.125	0.0625	0.03125
JSΔ*tolC-CL*	2	0.25	16	2	4	4	0.0625	0.0625
JSΔ*cpxA*Δ*tolC*	2	0.5	16	8	1	0.25	0.25	0.0625
JSc*pxA*_38_Δ*tolC*	4	1	16	4	1	0.25	0.25	0.625
JSc*pxA*_92__–__104_Δ*tolC*	2	1	16	4	1	0.25	0.5	0.125
JSΔ*cpxR*Δ*tolC*	0.125	0.03125	1	0.5	0.5	0.0625	0.03125	0.0157
JSΔ*ramA::Kan*	2	0.25	16	–	4	4	0.0625	0.0625
JSΔ*cpxA*Δ*ramA::Kan*	8	0.5	32	–	16	8	0.125	0.125
JSΔ*stm3031::Kan*	2	0.25	16	–	4	4	0.0625	0.0625
JSΔ*cpxA*Δ*stm3031::Kan*	8	0.5	32	–	32	16	0.25	0.25
JSΔ*htpX::Kan*	2	0.25	16	–	4	4	0.0625	0.0625
JSΔ*cpxA*Δ*htpX::Kan*	8	0.5	32	–	32	16	0.25	0.25
JSΔ*spy::Kan*	2	0.25	16	–	4	4	0.0625	0.0625
JSΔ*cpxA*Δ*spy::Kan*	8	0.5	32	–	32	16	0.25	0.25
JSΔ*acrD::Kan*	2	0.25	16	–	4	4	0.0625	0.0625
JSΔ*cpxA*Δ*acrD::Kan*	8	0.5	32	–	32	16	0.25	0.25
JSΔ*acrB*Δ*acrD::Kan*	0.5	0.25	4	–	0.5	0.125	0.0625	0.03125
JSΔ*cpxA*Δ*acrB*Δ*acrD::Kan*	2	1	16	–	1	0.5	0.25	0.0625

*AMK, amikacin; NET, netilmicin; STR, streptomycin; KAN, kanamycin; CEP, Cefalotin; CXM, cefuroxime; CRO, ceftriaxone; CTX, cefotaxime.*

### AGAs and β-Lactams Activate the CpxRA System

**FIGURE 1:**
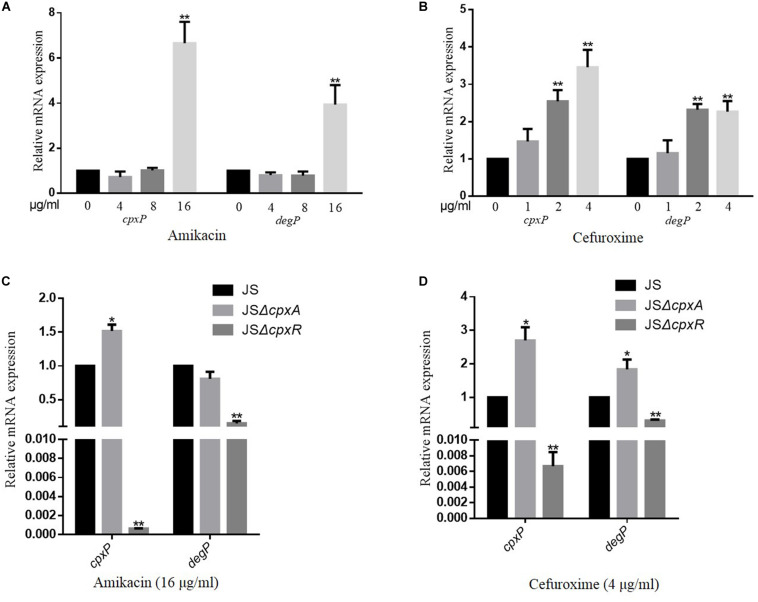
CpxRA is activated by amikacin and cefuroxime. **(A,B)** *S*. Typhimurium wild-type strains were grown in LB medium or LB medium with 4, 8, and 16 μg/ml amikacin or 1, 2, and 4 μg/ml cefuroxime to the stationary growth phase (OD_600_ ≥ 1.0), and mRNA levels of CpxRA-regulated genes (*cpxP* and *degP*) were measured by reverse transcription-qPCR. **(C,D)** *S*. Typhimurium wild-type strains, the JSΔ*cpxA*, or the JSΔ*cpxR* mutant strain were grown in LB medium with 16 μg/ml amikacin or 4 μg/ml cefuroxime to the stationary growth phase (OD_600_ ≥ 1.0), and mRNA levels *cpxP* and *degP* were measured by reverse transcription-qPCR. Data were normalized to *rpoD* expression levels. Bars represent means ± standard deviations. *, *P* < 0.05; **, *P* < 0.01.

To evaluate whether the CpxRA system is activated by AGAs and β-lactams, a series of concentrations of amikacin (4, 8, or 16 μg/ml) and cefuroxime (1, 2, or 4 μg/ml) were added to the culture medium. The expression levels of *cpxP* and *degP*, two target genes of the CpxRA system (Fujimoto et al., 2018), were measured using a qRT-PCR assay to evaluate the activation level of the CpxRA system of the wild-type strain JS in the presence or absence of the corresponding antimicrobial. As shown in Figures 1A,B, the transcription levels of both *cpxP* and *degP* increased with increasing amikacin and cefuroxime concentrations. Whereas deletion of *cpxR* resulted in significantly reduced expression levels of *cpxP* and *degP* in the presence of amikacin and cefuroxime, compared with the wild-type strain JS (Figures 1C,D). The deletion of *cpxA* resulted in significantly increased expression levels of *cpxP* in presence of cefuroxime and amikacin, increased expression levels of *degP* in presence of cefuroxime, and no significantly different expression levels of *degP* in presence of amikacin (Figures 1C,D), compared with the wild-type strain JS. These results suggest that AGAs and β-lactams activate the *S*. Typhimurium CpxRA system dependent on CpxR; however, *cpxA* deletion can activate the CpxRA system independently of amikacin and cefuroxime.

### AckA-Pta-Dependent Activation of CpxR Increases Antibiotic Resistance

To investigate the underlying genetic basis of the increased resistance of the *cpxA* deletion mutant to AGAs and β-lactams, the first step was to confirm whether the increased resistance of the *cpxA* null mutant was caused by pleiotropic effects, given that CpxA can regulate downstream target genes by interacting with other regulators rather than CpxR (Nakayama et al., 2003). For this reason, mutant strain JSΔ*cpxRA*, lacking both *cpxA* and *cpxR*, was constructed. The antibiotic sensitivity of the double mutant was equivalent to that of strain JSΔ*cpxR* (Table 2). We also constructed point mutations in *cpxR* (*cpxR*_*D*__51A_, *cpxR*_*M*__199A_) and corresponding double mutations based on the *cpxA* deletion mutations (Δ*cpxAcpxR*_*D*__51A_, Δ*cpxAcpxR*_*M*__199A_). Compared with strain JS, two- to four-fold decreases in the MICs of the tested AGA and β-lactam antibiotics for JS mutant *cpxR*_*D*__51A_ were observed, and there was no significant change in JS*cpxR*_*M*__199A_. Compared with strain JSΔ*cpxA*, 4- to 16-fold decreases in the MICs of the tested AGA and β-lactam antibiotics for mutant strains JSΔ*cpxAcpxR*_*D*__51A_ and JSΔ*cpxAcpxR*_*M*__199A_ were observed (Table 2). These results demonstrated that the observed resistance of the *cpxA* deletion mutant to AGAs and β-lactams is dependent on CpxR and is not caused by pleiotropic effects.

**FIGURE 2:**
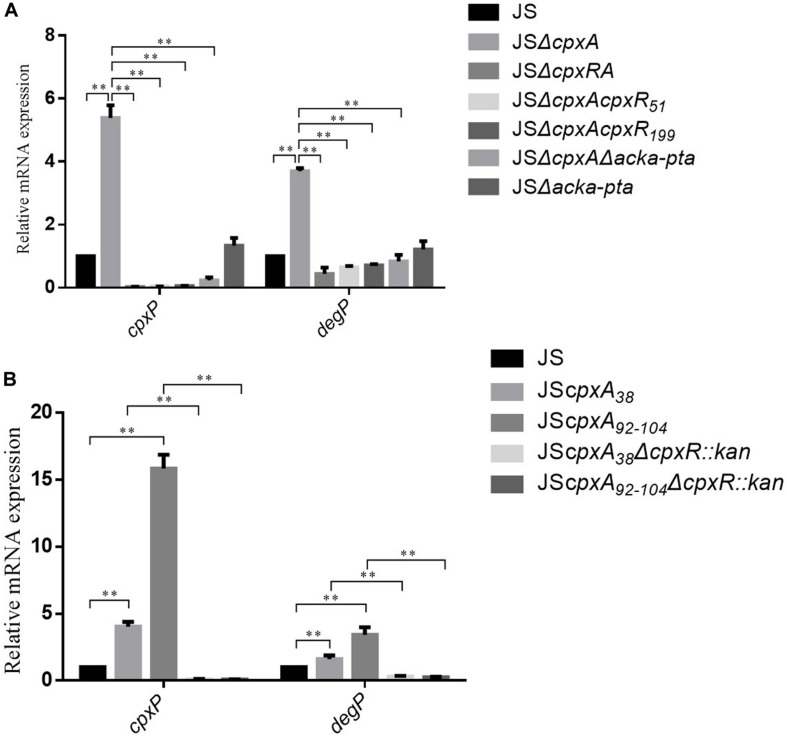
Relative mRNA levels of the CpxRA-regulated genes (*cpxP* and *degP*) were measured by real-time PCR. **(A)** Relative mRNA levels of the were measured in the WT *S*. Typhimurium JS strain and its isogenic JSΔ*cpxA*, JSΔ*cpxRA*, JSΔ*cpxAcpxR*_51_, JSΔ*cpxAcpxR*_199_, JSΔ*cpxA*Δ*acka-pta*, and JSΔ*acka-pta*. **(B)** Relative mRNA levels of the were measured in the WT *S*. Typhimurium JS strain and its isogenic JS*cpxA*_38_, JS*cpxA*_92__–__104_, JS*cpxA*_38_Δ*cpxR::kan*, JSΔ*cpxA*_92__–__104_Δ*cpxR::kan*. The expression level of each mRNA in strain JS represents one-fold. Data were normalized to *rpoD* expression levels. Bars represent means ± standard deviations. **, *P* < 0.01.

CpxA acts as an autokinase, a kinase that activates CpxR, and a phosphatase (7,8), and *cpxA* null mutations would therefore presumably disrupt all three of these activities. The lack of phosphatase activity leads to the phosphorylation of CpxR via alternative sources, such as acetyl phosphate produced by the AckA and Pta enzymes (14,28). To investigate whether AckA and Pta participate in the regulation of AGAs and β-lactams resistance in the *cpxA* deletion mutant, double deletion mutant JSΔ*ackA-pta* and triple deletion mutant JSΔ*cpxA*Δ*ackA-pta* were constructed. Compared with JSΔ*ackA-pta*, no significant differences in the MICs of any of the tested antibiotics against strain JSΔ*cpxA*Δ*ackA-pta* were observed. However, 2- to 16-fold decreases in the MICs of the tested antibiotics against JSΔc*pxA*Δ*ackA-pta* were observed compared with the MICs of JSΔ*cpxA* (Table 2). This result suggests that the increased resistance of the *cpxA* null mutant to AGA and β-lactam antibiotics is associated with AckA-Pta. We then evaluated the activation of the CpxRA system by measuring the expression levels of *cpxP* and *degP*. As shown in Figure 2A, compared with the wild-type strain, significant increases in mRNA levels of both genes were observed in strain JSΔ*cpxA*. Compared with strain JSΔ*cpxA*, significant decreases in the expression of both genes were observed in strains JSΔ*cpxRA*, JSΔ*cpxAcpxR*_*D*__51A_, JSΔ*cpxAcpxR*_*M*__199A_, and JSΔ*cpxA*Δ*ackA-pta*. It has been suggested that the knock-out of *cpxA* could confer the resistance to the AGA and β-lactam antibiotics. This regulatory mechanism is involved in the activation of the CpxRA system *via* phosphorylation of CpxR by the AckA-Pta pathway.

### AGAs and β-Lactams Resistance Can Be Conferred by CpxA-Mediated Ativation of CpxR

It has been confirmed that an in-frame deletion mutation that removed amino acids 92–104, or a site-directed mutation resulting in a Leu38Phe substitution in the periplasmic domain of CpxA, both constitutively activated the CpxRA system (collectively referred to as a *cpx*^∗^ mutation) (Pogliano et al., 1998; Humphreys et al., 2004). Here, to determine whether the CpxA-mediated activation of CpxR also confers AGAs and β-lactams resistance in *S*. Typhimurium, constitutively active mutants of *cpxA* (JS*cpxA*_38_ and JS*cpxA*_92__–__104_) were constructed and the MICs of AGAs and β-lactams were measured. Moreover, the corresponding complemented strains JS*cpxA*_38_-CL and JS*cpxA*_92__–__104_-*CL* were constructed. As shown in Table 2, the MICs of JS*cpxA*_38_ and JS*cpxA*_92__–__104_ were the same as that of the *cpxA* null mutant. Compared with strain JS*cpxA*_38_ and JS*cpxA*_92__–__104_, two- to eight-fold decreases in the MICs of the tested AGA and β-lactam antibiotics against the complemented strain JS*cpxA*_38_-CL and JS*cpxA*_92__–__104_-CL were observed. In the strains that do not produce *cpxR* (JSΔ*cpxA*_38_Δ*cpxR:kan*, JSΔ*cpxA*_92__–__104_Δ*cpxR:kan*), the MICs of all of the drugs except for kanamycin were the same as that of the *cpxR* deletion mutant (JSΔ*cpxR*). As shown in Figure 2B, compared with the wild-type strain, significant increases in mRNA levels of both target genes *cpxP* and *degP* were observed in strains JS*cpxA*_38_ and JS*cpxA*_92__–__104_, whereas significant decreases in the expression of both genes were observed in strains JS*cpxA*_38_Δc*pxR:kan* and JS*cpxA*_92__–__104_Δc*pxR:kan* compared with strains JS*cpxA*_38_ and JS*cpxA*_92__–__104_. These results demonstrate that resistance to AGAs and β-lactams can be conferred by activated CpxA, which is dependent on the phosphorylation of CpxR.

### CpxRA System Can Modulate Resistance to AGAs and β-Lactams Independent of the AcrAB-TolC Efflux Pump

**FIGURE 3:**
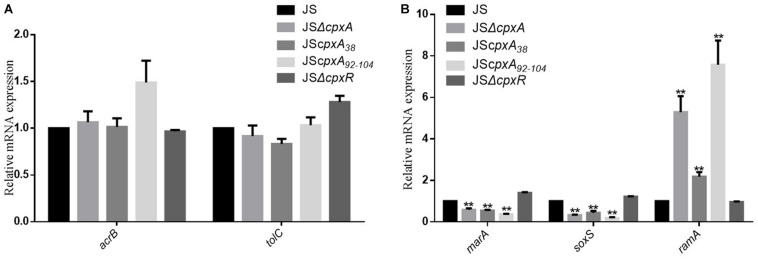
The influence of various *cpxA* mutations on the expression levels of the efflux pump AcrAB-TolC. **(A)** Relative mRNA expression levels of the efflux pump genes *acrB* and *tolC*. **(B)** Relative mRNA expression levels of the transcription factor genes *marA*, *soxS*, and *ramA*. The expression level of each mRNA in strain JS represents one-fold. Data were normalized to *rpoD* expression levels. Bars represent means ± standard deviations. **, *P* < 0.01.

Drug efflux mechanisms are ubiquitous among Gram-negative bacteria and contribute significantly to MDR. Among the mechanisms reported to date, the AcrAB-TolC efflux pump plays a significant role in resistance to various antibiotics (Baucheron et al., 2004; Du et al., 2014; Li et al., 2015). To investigate whether AcrAB-TolC affects CpxRA-mediated regulation of drug resistance, we constructed several double and triple deletion mutant strains from single mutants JSΔ*cpxA*, JS*cpxA*_38_, JS*cpxA*_92__–__104_, JSΔ*cpxR*, JSΔ*acrB*, and JSΔ*tolC*, as shown in Table 2. Compared with strain JS, 2- to 32-fold decreases in the MICs of all of the drugs except for netilmicin, kanamycin and ceftriaxone against strain JSΔ*acrB* were observed. Meanwhile, the MICs of all the tested antibiotics against complemented strain JSΔ*acrB-CL* were the same as strain JS. Compared with strain JSΔ*acrB*, two- to four-fold increases in the MICs of all of the drugs against strain JSΔ*cpxA*Δ*acrB*, JS*cpxA*_38_Δ*acrB*, and JS*CpxA*_92__–__104_Δ*acrB* were observed, whereas two- to four-fold decreases in the MICs of all of the drugs except for cefalotin against strain JSΔ*cpxR*Δ*acrB* were observed. Compared with strain JS, 2- to 32-fold decreases in the MICs of all of the drugs except for netilmicin and ceftriaxone against strain JSΔ*tolC* were observed. Meanwhile, the MICs of all the tested antibiotics against complemented strain JSΔ*tolC-CL* were the same as strain JS. Compared with strain JSΔ*tolC*, two- to eight-fold increases in the MICs of all of the drugs against strains JSΔ*cpxA*Δ*tolC*, JS*cpxA*_38_Δ*tolC*, and JS*cpxA*_92__–__104_Δ*tolC* were observed, whereas two- to eight-fold decreases in the MICs of all of the drugs except for cefalotin against strain JSΔ*cpxR*Δ*tolC* were observed (Table 2). As shown in Figure 3, the expression levels of *acrB*, *tolC*, and the transcription factor genes *marA*, *soxS*, and *ramA* were measured in strains JS, JSΔ*cpxA*, JS*cpxA*_38_, JS*cpxA*_92__–__104_, and JSΔ*cpxR*. In all *cpxA* mutants, a significant decrease in the expression levels of *marA* and *soxS* was observed, along with a significant increase in the expression level of *ramA*, and no significant difference in the expression levels of *acrB* and *tolC* were observed compared with strain JS. We then constructed mutants JSΔ*ramA::kan* and JSΔ*cpxA*Δ*ramA::kan*. Compared with strain JSΔ*ramA::kan*, two- to four-fold increases in the MICs of all of the drugs except for kanamycin against strain JSΔ*cpxA*Δ*ramA::kan* were observed. These results demonstrate that AcrB and TolC confer resistance to some AGAs and β-lactams, but both activation and inactivation of the CpxRA system can modulate resistance to AGAs and β-lactams in both an *acrB* or *tolC* background and a Δ*acrB* or Δ*tolC* background.

### Effects of Different CpxA Mutations on the Expression Levels of Cpx Regulons

**FIGURE 4:**
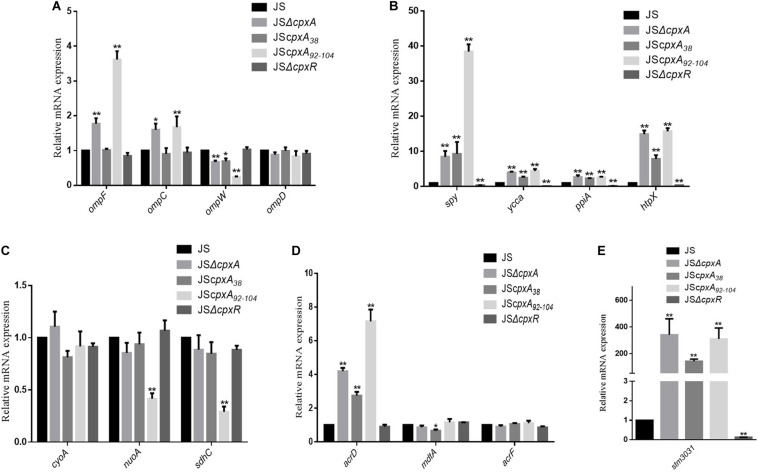
Relative mRNA levels of a series of MDR-related genes were measured by real-time PCR. **(A)** Relative mRNA expression levels of the outer membrane proteins genes *ompF*, *ompC*, *ompW*, and *ompD*; **(B)** Relative mRNA expression levels of protease genes *ppiA* and *htpX* and protein folding factors genes *spy* and *yccA*; **(C)** Relative mRNA expression levels of the genes involved in respiration *cyoA*, *nuoA*, and *sdhC*; **(D)** Relative mRNA expression levels of the efflux pumps genes *acrD*, *mdtA*, and *acrF*; **(E)** Relative mRNA expression levels of the outer membrane proteins genes STM3031; The expression level of each mRNA in strain JS represents one-fold. Data were normalized to *rpoD* expression levels. Bars represent means ± standard deviations. *, *P* < 0.05; **, *P* < 0.01.

The CpxRA system can alter the expression levels of a series of published Cpx regulon members to mediate drug resistance, such as those encoding outer membrane proteins OmpF, OmpC, OmpD, OmpW, and STM3031; efflux pump AcrD; proteases PpiA and HtpX; protein folding factors Spy and YccA, and respiration-related proteins CyoA, NuoA, and SdhC (Hu et al., 2011; Mahoney and Silhavy, 2013; Raivio et al., 2013; Raivio, 2014; Huang et al., 2016). To identify whether different *cpxA* mutations have a consistent effect on the expression levels of these Cpx regulons, we detected the relative mRNA expression levels of these genes. As shown in Figure 4, compared with strain JS, the mRNA expression levels of *ompW*, *acrD*, *spy*, *ycca*, *ppia*, *htpX*, and *stm3031* in all three *cpxA* mutants changed significantly (*P* < 0.01 or *P* < 0.05). In detail, the mRNA expression level of *ompW* was decreased, whereas the expression levels of *acrD*, *spy*, *ycca*, *ppia*, *htpX*, and *stm3031* were increased. The mRNA expression levels of *ompD*, *cyoA*, and *acrF* showed no significant differences in these three *cpxA* mutants. The mRNA expression levels of *ompF* and *ompC* were increased in JSΔ*cpxA* and JS*cpxA*_92__–__104_, but showed no significant difference in JS*cpxA*_38_. The mRNA expression levels of *nuoA* and *sdhC* were decreased in JS*cpxA*_92__–__104_, but showed no significant difference in JSΔ*cpxA* and JS*cpxA*_38_. In mutant strain JSΔ*cpxR*, significant decreases (*P* < 0.01 or *P* < 0.05) in mRNA expression were only seen for *spy*, *ycca*, *ppia*, *htpX*, and *stm3031*. Then, we individually constructed null mutations in sharply upregulated genes *stm3031*, *htpX*, and *spy* in a *cpxA*-deficient background and WT (JSΔ*stm3031::kan*, JSΔ*htpX::kan*, JSΔ*spy::kan*, JSΔ*cpxA*Δ*stm3031::kan*, JSΔ*cpxA*Δ*htpX::kan*, and JSΔ*cpxA*Δ*spy::kan*). However, compared with JSΔ*cpxA*, none of these genes showed a decrease in the resistance conferred by the *cpxA* deletion mutation (Table 2). To further study the impact of efflux pumps on the resistance conferred by the *cpxA* deletion mutation, we also constructed some corresponding *acrD* null mutations (JSΔ*acrD::Kan*, JSΔ*cpxA*Δ*acrD::Kan*, JSΔ*acrB*Δ*acrD::Kan*, and JSΔ*cpxA*Δ*acrB*Δ*acrD::Kan*). As shown in Table 2, the MICs of JSΔ*acrD::Kan* were the same as that of the JS. Compared with JSΔ*cpxA*, the MICs of JSΔ*cpxA*Δ*acrD::Kan* were the same as that of the *cpxA* null mutant. Compared with *JS*Δ*acrB*Δ*acrD::Kan*, two- to four-fold increases in the MICs of all of the drugs except for kanamycin against strain JSΔ*cpxA*Δ*acrB*Δ*acrD::Kan*.

## Discussion

In this study, all of the tested *cpxA* mutants of *S*. Typhimurium were found to confer resistance to AGAs and β-lactams. The previous studies showed the strain *cpxA*^∗^ (JS*cpxA*_92__–__104_) was more resistant to amikacin than its wild-type strain SL1344, but the *cpxA* deletion mutant was more sensitive to amikacin than its parent strain (Humphreys et al., 2004). Such contradictory findings suggested that although *cpxA* deletion can active the CpxRA system and display numerous phenotypes, whether null mutations of *cpxA* regulating resistance are caused solely by hyperphosphorylation of CpxR in *S*. Typhimurium still needs to be determined. On the basis of studies involving *cpxR* deletion and site-directed mutagenesis, we ruled out the pleiotropic effects of CpxA and provided evidence that the increased resistance of *cpxA* deletion mutants to AGAs and β-lactams is directly mediated by CpxR-P, which is activated *via* the acetyl phosphate pathway. This finding is consistent with previous reports showing that the deletion of *cpxA* eliminates its phosphatase activity, causing CpxR to accept phosphate groups from acetyl phosphate generated by Acka and Pta, activating the CpxRA system and contributing to some phenotypic changes (Danese and Silhavy, 1998; Buelow and Raivio, 2005; Lima et al., 2012).

JSΔ*cpxR* showed no significant differences in the mRNA expression levels of the major tested genes in the absence of antibiotics, which is consistent with a previous report in *S*. Typhimurium (Huang et al., 2016). It is also consistent with a previous study in *E. coli* that showed that a *cpxR* mutant had few differentially regulated genes compared with wild-type uropathogenic *E. coli*, which indicated that the CpxRA system is only minimally active in the wild-type strain under the experimental growth conditions (Dbeibo et al., 2018). This raises the question of why the wild-type strain shows more resistance to AGAs and β-lactams. Here, we confirmed that amikacin and cefuroxime can activate the CpxRA system and that the activated CpxRA system increases the resistance to amikacin and cefuroxime. Therefore, it is proposed that the presence of antibiotics activates the CpxRA response system in wild-type strains, which in turn, leads to an increase in drug resistance compared with strain JSΔ*cpxR*. The results also suggest that the phenotypic changes caused by *cpxR* deletion mainly depend on whether the CpxRA pathway can be activated by a corresponding external stimulation.

In Gram-negative bacteria, RND-family multidrug efflux pumps play the most prominent roles in drug resistance (Poole, 2001; Becker and Cooper, 2013; Du et al., 2018). Among the RND efflux pumps, AcrD is mainly responsible for resistance to the hydrophilic class of drugs such as AGAs in *E. coli* (Rosenberg et al., 2000). Moreover, some reports suggest that this efflux pump confers resistance to some other compounds, such as tetracycline, novobiocin, norfloxacin, Fosfomycin, and some β-lactams in *E. coli* (Nishino and Yamaguchi, 2001; Nishino et al., 2003). By contrast, AcrB confers resistance to practically all types of antibacterial agents, except aminoglycosides in *E. coli* (Elkins and Nikaido, 2002; Li et al., 2015). In the current study, a significant increase in the expression level of *acrD* was observed in the *cpxA* mutants, which is consistent with previous reports and supports the important role of the AcrAD-TolC efflux pump in resistance regulation of the CpxRA system (Hu et al., 2011; Huang et al., 2016). However, the *acrD* deletion mutation did not decrease the resistance conferred by the *cpxA* deletion mutation, which is consistent with previous research and suggests that no single factor is necessary of the resistance to β-lactams and AGAs conferred by the *cpxA* mutation (Mahoney and Silhavy, 2013). AcrAB-TolC contributes to the resistance to some aminoglycosides (amikacin and streptomycin) in *S. enterica* serovar Typhimurium JS, which is consistent with the previous result where the deletion of *acrB* decreased resistance to aminoglycosides (amikacin and neomycin) (Huang et al., 2016). To our knowledge, it is unclear why the mutant strain lacking *acrB* is more susceptible to some aminoglycosides in *S. enterica* serovar Typhimurium JS and a further study is needed. We did not find any significant change in the mRNA expression levels of *acrB* and *tolC*, this differed from a previous report showing that overexpression of *cpxR* results in a significant decrease in the mRNA expression levels of *acrB* and *tolC* compared with the wild-type strain JS (Huang et al., 2016). This difference may be explained by the paradigm that, for the tested global regulatory factors, the three *cpxA* mutations all led to a significant reduction in the levels of *marA* and *soxS*, thereby downregulating the expression levels of the AcrAB-TolC efflux pump, but led to a significant increase in the levels of *ramA*, thereby upregulating the expression of the AcrAB-TolC efflux pump. Moreover, in the *acrB* and *tolC* deletion mutants, which showed decreased resistance to AGAs and β-lactams, both activation and inactivation of the CpxRA system could modulate resistance. To investigate further, we constructed strain JSΔ*cpxA*Δ*ramA::kan* and JSΔ*ramA::kan*. Compared with strain JSΔ*ramA::kan*, the MICs of the tested antibiotics also showed an increase. These results suggest that the efflux pump AcrAB-TolC does not play a decisive role in the CpxRA-mediated AGAs and β-lactams resistance of *S*. Typhimurium.

Membrane proteases is one of the important mechanisms of AGAs resistance (Becker and Cooper, 2013). When bacteria are exposed to AGAs, there is an increase in the abundance of misfolded and mistargeted proteins within cells. Whereafter, genes that are involved in protein turnover are upregulated, as well as those encoding membrane proteases. If the membrane proteases cannot keep up with the increasing number of faulty proteins, their surplus accumulation eventually destroys the integrity of the membrane and kills the bacteria (Magnet and Blanchard, 2005; Becker and Cooper, 2013). In this study, the selected protease genes, *ppiA* and *htpX*, and the protein folding factor genes, *spy* and *yccA*, were all upregulated in various *cpxA* mutants, suggesting that the function of maintaining membrane integrity of the CpxRA envelope stress response system, is important for the resistance to AGAs.

One theory proposes that all bactericidal antibiotics may act through a common mechanism involving the production of reactive oxygen species (ROS), which is dependent on metabolism-related NADH depletion and the electron transport chain (Van Acker and Coenye, 2017). It has been shown that *cpxA* or *cpxR* deletion mutations confer antibiotic resistance by reducing the production of ROS (Kohanski et al., 2008). A study also demonstrated that compared with the parent strain, the expression levels of genes encoding succinate dehydrogenase (*sdh*), NADH dehydrogenase (*nuo*), and cytochrome oxidase (*cyo*) were significantly downregulated under multiple conditions in the transient NlpE overexpression strain, and the elimination of these genes (*cyoA*, *nuoA*, and *sdhC*) conferred resistance to amikacin (Raivio et al., 2013). However, we found that this common factor was inconsistent among the tested *cpxA* mutants. The mRNA expression levels of *nuoA* and *sdhC* were only found to be decreased in strain JS*cpxA*_92__–__104_, with no significant differences detected in strains JSΔ*cpxA* and JS*cpxA*_38_. These results suggested that the decrease in ROS may explain the accompanied tolerance to aminoglycosides of the activated CpxRA system in certain *cpxA* mutants, particularly JS*cpxA*_92__–__104_. Furthermore, the factors involved in drug resistance regulation appear to differ between the tested *cpxA* mutants.

## Conclusion

In conclusion, this study analyzed the mechanism involved in the CpxA-mediated regulation of resistance to AGAs and β-lactams in *S*. Typhimurium. It was revealed that various *cpxA* mutations show the same resistance phenotype relying on phosphorylated CpxR. These findings broaden our understanding of the complex regulatory network governing CpxRA-mediated antibiotic resistance.

## Data Availability Statement

The original contributions presented in the study are included in the article/Supplementary Material, further inquiries can be directed to the corresponding author/s.

## Author Contributions

WJ, JL, and SW designed the experiment. YL was responsible for funding acquisition and project supervision. WJ, XL, and YL contributed to manuscript writing. All authors contributed to the article and approved the submitted version.

## Conflict of Interest

The authors declare that the research was conducted in the absence of any commercial or financial relationships that could be construed as a potential conflict of interest.

## Supplementary Material

Supplementary Table 1 Primers used in this study.

## References

[B1] Baucheron S. Tyler S. Boyd D. Mulvey M. R. Chaslus-Dancla E. Cloeckaert A. (2004). AcrAB-TolC directs efflux-mediated multidrug resistance in *Salmonella enterica* serovar typhimurium DT104. *Antimicrob. Agents Chemother.* 48 3729–3735. 10.1128/AAC.48.10.3729-3735.2004 15388427 PMC521921

[B2] Becker B. Cooper M. A. (2013). Aminoglycoside antibiotics in the 21st century. *ACS Chem. Biol.* 8 105–115. 10.1021/cb3005116 23110460

[B3] Buelow D. R. Raivio T. L. (2005). Cpx signal transduction is influenced by a conserved N-terminal domain in the novel inhibitor CpxP and the periplasmic protease DegP. *J. Bacteriol.* 187 6622–6630. 10.1128/JB.187.19.6622-6630.2005 16166523 PMC1251582

[B4] Cockerill F. Wikler M. Alder J. Dudley M. Eliopoulos G. Ferraro M. (2012). Methods for dilution antimicrobial susceptibility tests for bacteria that grow aerobically: approved standard. *Clin. Lab. Stand. Inst.* 32 M07–M09.

[B5] Danese P. N. Silhavy T. J. (1998). CpxP, a stress-combative member of the Cpx regulon. *J. Bacteriol.* 180 831–839. 9473036 10.1128/jb.180.4.831-839.1998 PMC106961

[B6] Datsenko K. A. Wanner B. L. (2000). One-step inactivation of chromosomal genes in *Escherichia coli* K-12 using PCR products. *Proc. Natl. Acad. Sci. U. S. A.* 97 6640–6645. 10.1073/pnas.120163297 10829079 PMC18686

[B7] Dbeibo L. van Rensburg J. J. Smith S. N. Fortney K. R. Gangaiah D. Gao H. (2018). Evaluation of CpxRA as a Therapeutic Target for Uropathogenic *Escherichia coli* Infections. *Infect. Immun.* 86 798–717e. 10.1128/IAI.00798-717 PMC5820967 29311237

[B8] De la Cruz M. A. Perez-Morales D. Palacios I. J. Fernandez-Mora M. Calva E. Bustamante V. H. (2015). The two-component system CpxR/A represses the expression of *Salmonella* virulence genes by affecting the stability of the transcriptional regulator HilD. *Front. Microbiol.* 6:807 10.3389/fmicb.2015.00807 PMC4526804 26300871

[B9] De Wulf P. McGuire A. M. Liu X. Lin E. C. (2002). Genome-wide profiling of promoter recognition by the two-component response regulator CpxR-P in *Escherichia coli*. *J. Biol. Chem.* 277 26652–26661. 10.1074/jbc.M203487200 11953442

[B10] Delhaye A. Collet J. F. Laloux G. (2016). Fine-Tuning of the Cpx Envelope Stress Response Is Required for Cell Wall Homeostasis in *Escherichia coli*. *mBio* 7 47–16e. 10.1128/mBio.00047-16 PMC4791840 26908573

[B11] DiGiuseppe P. A. Silhavy T. J. (2003). Signal detection and target gene induction by the CpxRA two-component system. *J. Bacteriol.* 185 2432–2440. 10.1128/jb.185.8.2432-2440.2003 12670966 PMC152615

[B12] Du D. Wang-Kan X. Neuberger A. van Veen H. W. Pos K. M. Piddock L. J. V. (2018). Multidrug efflux pumps: structure, function and regulation. *Nat. Rev. Microbiol.* 16 523–539. 10.1038/s41579-018-0048-46 30002505

[B13] Du D. Wang Z. James N. R. Voss J. E. Klimont E. Ohene-Agyei T. (2014). Structure of the AcrAB-TolC multidrug efflux pump. *Nature* 509 512–515. 10.1038/nature13205 24747401 PMC4361902

[B14] Elkins C. A. Nikaido H. (2002). Substrate specificity of the RND-type multidrug efflux pumps AcrB and AcrD of *Escherichia coli* is determined predominantly by two large periplasmic loops. *J. Bacteriol.* 184 6490–6498. 10.1128/jb.184.23.6490-6499.2002 12426336 PMC135441

[B15] Fujimoto M. Goto R. Haneda T. Okada N. Miki T. (2018). *Salmonella enterica* Serovar Typhimurium CpxRA Two-Component System Contributes to Gut Colonization in *Salmonella*-Induced Colitis. *Infect. Immun.* 86 280–218e. 10.1128/IAI.00280-218 PMC6013652 29685984

[B16] Gotoh Y. Eguchi Y. Watanabe T. Okamoto S. Doi A. Utsumi R. (2010). Two-component signal transduction as potential drug targets in pathogenic bacteria. *Curr. Opin. Microbiol.* 13 232–239. 10.1016/j.mib.2010.01.008 20138000

[B17] Hirakawa H. Nishino K. Hirata T. Yamaguchi A. (2003a). Comprehensive studies of drug resistance mediated by overexpression of response regulators of two-component signal transduction systems in *Escherichia coli*. *J. Bacteriol.* 185 1851–1856. 12618449 10.1128/JB.185.6.1851-1856.2003 PMC150137

[B18] Hirakawa H. Nishino K. Yamada J. Hirata T. Yamaguchi A. (2003b). Beta-lactam resistance modulated by the overexpression of response regulators of two-component signal transduction systems in *Escherichia coli*. *J. Antimicrob. Chemother.* 52 576–582. 10.1093/jac/dkg406 12951338

[B19] Hu W. S. Chen H. W. Zhang R. Y. Huang C. Y. Shen C. F. (2011). The expression levels of outer membrane proteins STM1530 and OmpD, which are influenced by the CpxAR and BaeSR two-component systems, play important roles in the ceftriaxone resistance of *Salmonella enterica* serovar Typhimurium. *Antimicrob. Agents Chemother.* 55 3829–3837. 10.1128/AAC.00216-211 21646491 PMC3147640

[B20] Huang H. Sun Y. Yuan L. Pan Y. Gao Y. Ma C. (2016). Regulation of the Two-Component Regulator CpxR on Aminoglycosides and beta-lactams Resistance in *Salmonella enterica* serovar Typhimurium. *Front. Microbiol.* 7:604 10.3389/fmicb.2016.00604 PMC4846824 27199934

[B21] Humphreys S. Rowley G. Stevenson A. Anjum M. F. Woodward M. J. Gilbert S. (2004). Role of the two-component regulator CpxAR in the virulence of *Salmonella enterica* serotype Typhimurium. *Infect. Immun.* 72 4654–4661. 10.1128/IAI.72.8.4654-4661.2004 15271926 PMC470642

[B22] Isaac D. D. Pinkner J. S. Hultgren S. J. Silhavy T. J. (2005). The extracytoplasmic adaptor protein CpxP is degraded with substrate by DegP. *Proc. Natl. Acad. Sci. U. S. A.* 102 17775–17779. 10.1073/pnas.0508936102 16303867 PMC1308919

[B23] Jing W. Liu J. Wu S. Chen Q. Li X. Liu Y. (2020). Development of a Method for Simultaneous Generation of Multiple Genetic Modification in *Salmonella enterica* Serovar Typhimurium. *Front. Genet.* 11:563491 10.3389/fgene.2020.563491 PMC7544003 33193646

[B24] Kohanski M. A. Dwyer D. J. Wierzbowski J. Cottarel G. Collins J. J. (2008). Mistranslation of membrane proteins and two-component system activation trigger antibiotic-mediated cell death. *Cell* 135 679–690. 10.1016/j.cell.2008.09.038 19013277 PMC2684502

[B25] Levy S. B. (2002). The 2000 Garrod lecture. *Factors impacting on the problem of antibiotic resistance*. *J. Antimicrob. Chemother.* 49 25–30. 10.1093/jac/49.1.25 11751763

[B26] Li X. Z. Plesiat P. Nikaido H. (2015). The challenge of efflux-mediated antibiotic resistance in Gram-negative bacteria. *Clin. Microbiol. Rev.* 28 337–418. 10.1128/CMR.00117-114 25788514 PMC4402952

[B27] Lima B. P. Thanh Huyen T. T. Basell K. Becher D. Antelmann H. Wolfe A. J. (2012). Inhibition of acetyl phosphate-dependent transcription by an acetylatable lysine on RNA polymerase. *J. Biol. Chem.* 287 32147–32160. 10.1074/jbc.M112.365502 22829598 PMC3442545

[B28] Magnet S. Blanchard J. S. (2005). Molecular insights into aminoglycoside action and resistance. *Chem. Rev.* 105 477–498. 10.1021/cr0301088 15700953

[B29] Mahoney T. F. Silhavy T. J. (2013). The Cpx stress response confers resistance to some, but not all, bactericidal antibiotics. *J. Bacteriol.* 195 1869–1874. 10.1128/JB.02197-2112 23335416 PMC3624577

[B30] Masi M. Pinet E. Pages J. M. (2020). Complex Response of the CpxAR Two-Component System to beta-Lactams on Antibiotic Resistance and Envelope Homeostasis in *Enterobacteriaceae*. *Antimicrob. Agents Chemother.* 64:32229490 10.1128/AAC.00291-220 PMC7269474 32229490

[B31] Nakayama S. Kushiro A. Asahara T. Tanaka R. Hu L. Kopecko D. J. (2003). Activation of hilA expression at low pH requires the signal sensor CpxA, but not the cognate response regulator CpxR, in *Salmonella enterica* serovar Typhimurium. *Microbiology* 149 2809–2817. 10.1099/mic.0.26229-26220 14523114

[B32] Nishino K. Yamada J. Hirakawa H. Hirata T. Yamaguchi A. (2003). Roles of TolC-dependent multidrug transporters of *Escherichia coli* in resistance to beta-lactams. *Antimicrob. Agents Chemother.* 47 3030–3033. 10.1128/aac.47.9.3030-3033.2003 12937021 PMC182617

[B33] Nishino K. Yamaguchi A. (2001). Analysis of a complete library of putative drug transporter genes in *Escherichia coli*. *J. Bacteriol.* 183 5803–5812. 10.1128/JB.183.20.5803-5812.2001 11566977 PMC99656

[B34] Nishino K. Yamasaki S. Hayashi-Nishino M. Yamaguchi A. (2010). Effect of NlpE overproduction on multidrug resistance in *Escherichia coli*. *Antimicrob. Agents Chemother.* 54 2239–2243. 10.1128/AAC.01677-1679 20211889 PMC2863614

[B35] Padilla-Vaca F. Mondragon-Jaimes V. Franco B. (2017). General Aspects of Two-Component Regulatory Circuits in Bacteria: Domains. *Sign. Roles. Curr. Prot. Pept. Sci.* 18 990–1004. 10.2174/1389203717666160809154809 27514854

[B36] Pogliano J. Dong J. M. De Wulf P. Furlong D. Boyd D. Losick R. (1998). Aberrant cell division and random FtsZ ring positioning in *Escherichia coli* cpxA^∗^ mutants. *J. Bacteriol.* 180 3486–3490. 10.1128/JB.180.13.3486-3490.1998 9642209 PMC107311

[B37] Poole K. (2001). Multidrug resistance in Gram-negative bacteria. *Curr. Opin. Microbiol.* 4 500–508. 11587924 10.1016/s1369-5274(00)00242-3

[B38] Quinn T. O’Mahony R. Baird A. W. Drudy D. Whyte P. Fanning S. (2006). Multi-drug resistance in *Salmonella enterica*: efflux mechanisms and their relationships with the development of chromosomal resistance gene clusters. *Curr. Drug Targets* 7 849–860. 16842216 10.2174/138945006777709548

[B39] Raivio T. L. (2014). Everything old is new again: an update on current research on the Cpx envelope stress response. *Biochim. Biophys. Acta* 1843 1529–1541. 10.1016/j.bbamcr.2013.10.018 24184210

[B40] Raivio T. L. Leblanc S. K. Price N. L. (2013). The *Escherichia coli* Cpx envelope stress response regulates genes of diverse function that impact antibiotic resistance and membrane integrity. *J. Bacteriol.* 195 2755–2767. 10.1128/JB.00105-113 23564175 PMC3697260

[B41] Raivio T. L. Silhavy T. J. (1997). Transduction of envelope stress in *Escherichia coli* by the Cpx two-component system. *J. Bacteriol.* 179 7724–7733. 9401031 10.1128/jb.179.24.7724-7733.1997 PMC179735

[B42] Rosenberg E. Y. Ma D. Nikaido H. (2000). AcrD of *Escherichia coli* is an aminoglycoside efflux pump. *J. Bacteriol.* 182 1754–1756. 10692383 10.1128/jb.182.6.1754-1756.2000 PMC94475

[B43] Tiwari S. Jamal S. B. Hassan S. S. Carvalho P. Almeida S. Barh D. (2017). Two-Component Signal Transduction Systems of Pathogenic Bacteria As Targets for Antimicrobial Therapy: An Overview. *Front. Microbiol.* 8:1878 10.3389/fmicb.2017.01878 PMC5641358 29067003

[B44] Van Acker H. Coenye T. (2017). The Role of Reactive Oxygen Species in Antibiotic-Mediated Killing of Bacteria. *Trends Microbiol.* 25 456–466. 10.1016/j.tim.2016.12.008 28089288

[B45] Wolfe A. J. Parikh N. Lima B. P. Zemaitaitis B. (2008). Signal integration by the two-component signal transduction response regulator CpxR. *J. Bacteriol.* 190 2314–2322. 10.1128/JB.01906-1907 18223085 PMC2293188

