# Supplementary material for: Role of cpxA Mutations in the Resistance to Aminoglycosides and β-Lactams in Salmonella enterica serovar Typhimurium
Source: Front Microbiol. 2021 Feb 4;12:604079. doi: 10.3389/fmicb.2021.604079 (PMC7889517; doi:10.3389/fmicb.2021.604079)
Supplement: Supplementary Table 1 — Primers used in this study. [file Table_1.DOCX]

**TABLE S1** **Primers used in this study**

| Primer | 5’ to 3’ sequence | Sources |
| --- | --- | --- |
| **FOR GENE DELETIONS** | | |
| cpxA-H1P1 | CTATCTGATGGTTTCCGCTTCATG  ATAGGAAGTTTAACCGCGCGCG  TGTAGGCTGGAGCTGCTTC | This study |
| cpxA-H2P2 | CCTGCATTCGCAGGCCGATGGT  TTTTAGGTTCGCTTGTACAGCGG  CATATGAATATCCTCCTTAG |  |
| cpxR-H1P1 | GATGATGACCGAGAGCTGACTTC  CCTGTTAAAAGAGCTCCTCGAA  GTGTAGGCTGGAGCTGCTTC | This study |
| cpxR-H2P2 | CAATGTTTTAAACCACGGGTGA  CCGTCTTTGCGTTCCGGCAGTTT  CATATGAATATCCTCCTTAG |  |
| ackA-pta-H1P1 | GCCACGTATCATAAATAGGTACTT  CCATGTCGAGTAAGTTAGTACTG  GTTGTGTAGGCTGGAGCTGCTTC | This study |
| ackA-pta-H2P2 | CCGCCATCCGGCATTAGCTTTTA  CTGTTACTGCTGCTGCTGAGAAG  CCTGCATATGAATATCCTCCTTAG |  |
| acrB-H1P1 | TGATCAACCTGCTCAGCCCAGGTC  TTAACTTAAACAGGAGCCGTTAA  GACGTGTAGGCTGGAGCTGCTTC | This study |
| acrB-H2P2 | TCATACAATGCCGCCAGACACAG  GAAGACGACGATCAGCGATATAG  CATACATATGAATATCCTCCTTAG |  |
| TolC-H1P1 | ATTTTTACAAATTGATCAGCGCTA  AATACTGCTTCACAACAAGGAGT  GTAGGCTGGAGCTGCTTC | This study |
| TolC-H2P2 | TACAAGGGCACAGGTCTGATAA  GCGCAGCGCCAGCGAATAACTT  ACATATGAATATCCTCCTTAG |  |
| ramA-H1P1 | AAATGTGCGGTGCGGGAGCCGC  TGACGAGTTTGATAGAGGGGAGA  GCACGGTGTAGGCTGGAGCTGCTTC | This study |
| ramA-H2P2 | ACATTTCAATGCGTACGGCCATG  CTTTTCTTTACGATAAGCGCCTGG  CGGCATATGAATATCCTCCTTAG |  |
| stm3031-H1P1 | ACTTAATACATGCCAGTAAGGTATC  AGGTAAAATAACCGGAGAGAAGAA  GGTGTAGGCTGGAGCTGCTTC | This study |
| stm3031-H2P2 | ATCGGATTATCCTATTCAGAACC  GGTAACCCACGCCAAGATTAAA  ACCATCATATGAATATCCTCCTTAG |  |
| htpX-H1P1 | GTAATCGCATAGTGCGCTTTGTT  AAATTGAGGTTAAAAGAAAATT  GTGTAGGCTGGAGCTGCTTC | This study |
| htpX-H2P2 | GTGGGCTTATTATTTCAGGTACT  CGCCGCTACGCAGCGCTTCAAT  CATATGAATATCCTCCTTAG |  |
| spy-H1P1 | TAACTGAAAGGAAGGATAGAAA  TATGCGTAAACTGACTGCTCTATT  TGTTGTGTAGGCTGGAGCTGCTTC | This study |
| spy-H2P2 | TCAACGATTGTGTTGTCGACAAG  ACCGGCGGTCTTAAATTATGCGGA  AAGCATATGAATATCCTCCTTAG |  |
| acrD-H1P1 | ACAGTGAAGCAGTTCAAATCTATAACGATATGTAGAAACACGAGGTTTCCCTTTACGCTGCCGCAAGCACTCA | This study |
| acrD-H2P2 | GTGCCCGACACCTCGTATCAGGCTGGCCGGGATCTTATTTCGGGCGCGGCTTCAGGCTTTTGAAGCTGGGGTGGG |  |
| **FOR COMPLEMENTING THE MUTATIONS** | | |
| CpxA-F | TTCGGGCTGCAAACATGC | (Jing et al., 2020) |
| CpxA-R | GGCTGGCTGTATCGGGTCA |  |
| CpxA*-F | GGTTTAAAACATTGCGTGGTCG | (Jing et al., 2020) |
| CpxA*-R | GTGCCGGAAATCTCCCGGTA |  |
| CpxA-UF | ATTCGAGCTCGGTACCCGGGCGAAATGGAAGGTTTTAATGTC | (Jing et al., 2020) |
| CpxA-DR | CCTGCAGGTCGACTCTAGAGCGAGTTTGATGCGGTGCAG |  |
| CpxA-CL-UR | CCGATGGTTT*CTA*GGTTCGCTTGTACAGCGGTA | (Jing et al., 2020) |
| CpxA-CL-DF | CAAGCGAACC*TAG*AAACCATCGGCCTGCGAATG |  |
| sacBKan-K1 | GCCTGGACGTTTGGGACA | (Jing et al., 2020) |
| sacBKan-K2 | CGGATAAAATGCTTGATGGTCG |  |
| AcrB-CL-F | CAAACAGCAAGCCGCAAGCGGTGATCAAC  CTGCTCAGCCCAGGTCTTAACTTAAACAGG  AGCCGTTAAGAC | This study |
| AcrB-CL-R | GGAGAACGGGATAGACCAGCTCTCATACAA  TGCCGCCAGACACAGGAA*CAC*GACGATCAG  CGATATAGCATA |  |
| TolC-CL-F | TGGCGGATTCTGCTAGAATCAGCAATTATTT  TTACAAATTGATCAGCGCTAAATACTGCTTC  ACAACAAGGA | This study |
| TolC-CL-R | AGACCTACAAGGGCACAGGTCTGATAAGC  GCAGCGCCAGCGAATAACTTA*TTA*ATGCCG  GAATGGATTGCCG |  |
| **REAL-TIME RELATIVE QUANTITATIVE PCR** | | |
| q-ropD-F | CGATCTTATCACCGGCTTTGT | (Fujimoto et al., 2018) |
| q-ropD-R | TTCTTCATCTTCGTCTTCGTCATC |  |
| q-cpxP-F | AGCGTAGCGCGCAAAATC | (Fujimoto et al., 2018) |
| q-cpxP-R | CTGTTCGTGCCTTGCCTGT |  |
| q-degP-F | GCGTAAATTCGATGCTAAAGTGG | (Fujimoto et al., 2018) |
| q-degP-R | ACCGTTTCGCCCAGACCA |  |
| q-ompF-F | CCTGGCAGCGGTGATCC | (Tatavarthy and Cannons, 2010) |
| q-ompF-R | AAATTTCTGCTGCGTTTGCG |  |
| q-ompC-F | TCGCAGCCTGCTGAACCAGAAC | (Hu et al., 2011) |
| q-ompC-R | ACGGGTTGCGTTATAGGTCTGAG |  |
| q-ompD-F | GCAACCGTACTGAAAGCCAGGG | (Hu et al., 2011) |
| q-ompD-R | GCCAAAGAAGTCAGTGTTACGGT |  |
| q-ompW-F | CAGCAGCAAAGTGCGTCCTTATGT | (Hu et al., 2011) |
| q-ompW-R | AGACAGAGGCGCCAATTAACCAGT |  |
| q-stm3031-F | TGCAAGCAGGGAGTAATAACGGGT | (Hu et al., 2011) |
| q-stm3031-R | TCACTTGGATACGCCCAGTCCCAT |  |
| q-acrD-F | TCCGGCCAAATTGAATAGTT | (Eaves et al., 2004) |
| q-acrD-R | TCGGAACCGTCCTGATTAAC |  |
| q-acrF-F | TATCTGGCTGGATGCGAATCTGCT | (Eaves et al., 2004) |
| q-acrF-R | ACTTTGCCGAACTCTTCCGGATCT |  |
| q-mdtA-F | GAATGCGCGTCGTGATCTG | (Nishino et al., 2007) |
| q-mdtA-R | TCCAGTTCCTGACGGGAAAC |  |
| q-tolC-F | GCCCGTGCGCAATATGAT | (Nishino et al., 2007) |
| q-tolC-R | CCGCGTTATCCAGGTTGTTG |  |
| q-acrB-F | CGTGAGCGTTGAGAAGTCCT | (Li et al., 2009) |
| q-acrB-R | GGCGTCAGTTGGTATTTGGT |  |
| q-marA-F | ATACATCCGCAGCCGTAAAA | (Li et al., 2009) |
| q-marA-R | GTGATTCGCCATGCATATTG |  |
| q-soxS-F | TACGGTAACGCATCAAACA | (Li et al., 2009) |
| q-soxS-R | ACAGGCGGTGACGGTAAT |  |
| q-ramA-F | CACGATTGTCGAGTGGATTG | This study |
| q-ramA-R | AAAATGCGCGTAAAGGTTTG |  |
| q-cyoA-F | GGTCCCGTGGAATTGAGGTC | This study |
| q-cyoA-R | TCCAGCAGCGCAGAATTACA |  |
| q-nuoA-F | CGCCCATCTGCCGTAAAGAG | This study |
| q-nuoA-R | CCAAAAACCAACCGCCTACC |  |
| q-sdhC-F | TAAGAACAGCATGTGGGCGT | This study |
| q-sdhC-R | GCGATGGAGAATGGACGCTA |  |

Italic letters indicate the mutation site in the primer. The overlapping sequence is underlined.

**REFERENCES**

Eaves, D.J., Ricci, V., and Piddock, L.J. (2004). Expression of acrB, acrF, acrD, marA, and soxS in Salmonella enterica serovar Typhimurium: role in multiple antibiotic resistance. *Antimicrob Agents Chemother* 48(4)**,** 1145-1150. doi: 10.1128/aac.48.4.1145-1150.2004.

Fujimoto, M., Goto, R., Haneda, T., Okada, N., and Miki, T. (2018). Salmonella enterica Serovar Typhimurium CpxRA Two-Component System Contributes to Gut Colonization in Salmonella-Induced Colitis. *Infect Immun* 86(7). doi: 10.1128/IAI.00280-18.

Hu, W.S., Chen, H.W., Zhang, R.Y., Huang, C.Y., and Shen, C.F. (2011). The expression levels of outer membrane proteins STM1530 and OmpD, which are influenced by the CpxAR and BaeSR two-component systems, play important roles in the ceftriaxone resistance of Salmonella enterica serovar Typhimurium. *Antimicrob Agents Chemother* 55(8)**,** 3829-3837. doi: 10.1128/AAC.00216-11.

Jing, W., Liu, J., Wu, S., Chen, Q., Li, X., and Liu, Y. (2020). Development of a Method for Simultaneous Generation of Multiple Genetic Modification in Salmonella enterica Serovar Typhimurium. *Front Genet* 11**,** 563491. doi: 10.3389/fgene.2020.563491.

Li, L., Wang, Y. P., Wu, Y. N., Wu, C. M., and Shen, J. Z. (2009). Association of marR, soxR and acrR mutations with multiple antibiotic resistance in ciprofloxacin-selected *Salmonella* mutants. *Food Saf. Qual. Detect. Technol.* 1,1–9.

Nishino, K., Nikaido, E., and Yamaguchi, A. (2007). Regulation of multidrug efflux systems involved in multidrug and metal resistance of Salmonella enterica serovar Typhimurium. *J Bacteriol* 189(24)**,** 9066-9075. doi: 10.1128/JB.01045-07.

Tatavarthy, A., and Cannons, A. (2010). Real-time PCR detection of Salmonella species using a novel target: the outer membrane porin F gene (ompF). *Lett Appl Microbiol* 50(6)**,** 645-652. doi: 10.1111/j.1472-765X.2010.02848.x.
